# The effect of probiotic and synbiotic supplementation on sleep parameters in exercised population: a systematic review and synthesis without meta-analysis (SWiM) of randomized controlled trials

**DOI:** 10.1080/15502783.2026.2670564

**Published:** 2026-05-25

**Authors:** Mina Salehi Asl, Fatemeh Ahmadi, Mohamadjavad Ershadmanesh, Erfan Mortezapour, Fereshteh Torki, Arshiya Danandeh, Elaheh Dehghani, Khashayar Danandeh

**Affiliations:** a School of Medicine, Shahid Beheshti University of Medical Sciences, Tehran, Iran; b Sports Medicine Research Center (SMRC), Neuroscience Institute, Tehran University of Medical Sciences (TUMS), Tehran, Iran; c Faculty of Mechanics, Electrical Power and Computer, Islamic Azad University, Science and Research Branch, Tehran, Iran

**Keywords:** Probiotic, synbiotic, sleep, sports nutrition, exercise, systematic review, narrative synthesis

## Abstract

**Background:**

Sleep is crucial for recovery and optimal performance in athletes; however, poor sleep is common during periods of intensive training or competition. The microbiota–gut–brain axis suggests probiotics and synbiotics could modulate sleep, but evidence in exercised populations is limited.

**Objective:**

To systematically review randomized controlled trials (RCTs) assessing the effects of probiotic and synbiotic supplementation on sleep in exercised populations, using a Synthesis Without Meta-analysis (SWiM) approach.

**Methods:**

PubMed, Scopus, Web of Science, and ProQuest were searched up to September 2025. Eligible studies were RCTs including exercised individuals, testing probiotics or synbiotics, and reporting sleep outcomes. Data extraction, risk of bias assessment (RoB 2), and narrative synthesis followed SWiM guidelines.

**Results:**

Six RCTs (*n* = 180) were included: four probiotic and two synbiotic interventions lasting 4–17 weeks. Populations included athletes from multiple sports across four continents. Nine of twelve primary sleep outcomes favored supplementation, with significant effects for probiotics (combined *p* < 0.01) and synbiotics (*p* < 0.001). Benefits were most consistent for subjective sleep quality and, in some cases, sleep latency. Secondary outcomes showed occasional reductions in stress, anxiety, and fatigue.

**Conclusion:**

Probiotic and synbiotic supplementation may improve sleep in exercised populations, especially perceived quality and latency. Evidence supports cautious, adjunctive use, but larger, standardized trials are needed to confirm effects.

PROSPERO registration number: CRD420251151264.

## 1. Introduction

Sleep is a critical determinant of recovery, adaptation, and performance in physically active individuals and athletes [1,2]. Yet, insufficient and poor-quality sleep are prevalent in athletic populations, particularly during intensive training or competition phases [3,4]. Studies indicate that athletes report poor sleep quality, with sleep durations of less than 7 hours per night [3]. These findings highlight a significant concern, as sleep duration and quality are crucial for recovery and performance, especially in sports. Recent consensus emphasises that sleep disturbances are predictable in athletes, particularly during periods of heightened stress, such as training cycles or travelling for competitions [5,6].

Growing attention has therefore turned to biology that might be leveraged to support sleep without compromising performance. The microbiota–gut–brain axis provides a plausible framework: microbially derived metabolites and signalling (e.g. serotonin/melatonin) interface with immune, neuroendocrine, and autonomic pathways relevant to sleep architecture and circadian rhythmicity [7–10]. Reviews highlight that specific stressors coincide with periods of heightened sleep vulnerability, precisely where adjunct nutrition strategies could be valuable [11].

Within this context, probiotics and synbiotics are increasingly studied for sleep outcomes in the general population. A 2024 meta-analysis of randomised, placebo-controlled trials found that probiotics reduced Pittsburgh Sleep Quality Index (PSQI) scores at 4–6 and 8–16 weeks and delivered small but significant gains in sleep efficiency; effects on sleep duration and insomnia severity were less consistent, and risk-of-bias/potential publication bias were noted [12].

Evidence specific to exercise populations remains limited and heterogeneous [13–18]. In a 17-week double-blind RCT in elite rugby union athletes spanning domestic and international competition, a multispecies probiotic programme was associated with favourable changes in self-reported sleep quality, with exploratory links to CRP and melatonin dynamics across the season [14]. Other athlete and performer trials (e.g. high-stress dancers) have included sleep as a prespecified outcome, but small samples and diverse measures hinder cross-study comparisons [18].

Despite high and predictable sleep burden in athletes, there has been no focused, transparent synthesis of randomised trials testing probiotic or synbiotic supplementation on sleep specifically in exercised populations that can guide practice. The current literature uses varied strains/mixtures (probiotics vs. synbiotic), dosing and durations, and crucially non-uniform sleep endpoints (PSQI, OSA inventory, actigraphy metrics, athlete-specific tools). Given the substantial heterogeneity and the small number of comparable trials, a conventional meta-analysis may be misleading or inapplicable. Therefore, we prespecified a Synthesis Without Meta-analysis (SWiM) approach to transparently group studies and synthesise findings across RCTs in exercised populations in line with SWiM reporting guidance. To systematically review randomised controlled trials of probiotic and synbiotic supplementation assessing sleep parameters in exercised populations and synthesise findings using a SWiM framework to inform future research and practice.

## 2. Method and materials

The review was registered in PROSPERO (CRD420251151264) and conducted following the methodological guidance of the Cochrane Handbook for Systematic Reviews of Interventions [19] and we adhered to the PRISMA 2020 (Preferred Reporting Items for Systematic Reviews and Meta-Analyses) reporting standards [20].

Two authors working independently (FA and ME) searched PubMed, Scopus, and Web of Science up to September 2025 to identify eligible studies, and also explored grey literature using ProQuest dissertations and theses. A detailed search strategy was developed based on the population-intervention-comparison-outcome-study design (PICOS) framework to ensure the comprehensive identification of relevant studies. The population (*P*) consisted of athletes or exercised individuals. The intervention (I) involved probiotics and synbiotic supplements. Comparators (C) included the standard of care, placebo, or control group. The outcomes (O) evaluated parameters related to sleep. Eligible study designs (S) included RCTs. Additional details on the PICOS framework are provided in **Supplementary File 1**. Language and publication-date restrictions were not applied. No non-English articles met the eligibility criteria and were included. On completion of the searches, bibliographies of relevant articles were hand-searched to capture additional records. Study records were organised in EndNote (X9.3.3), and duplicate citations were identified and eliminated using EndNote’s built-in detection tool.

### 2.1. Selection of studies

Inclusion criteria were: (a) randomised controlled design (RCT); (b) populations comprising athletes or individuals engaged in exercise; (c) original studies examining short- or long-term effects of probiotic and synbiotic supplementation; and (d) sufficient reporting of sleep-related outcomes. Exclusion criteria comprised: (a) in vitro, in silico, or in vivo animal experiments; and (b) ecological, cross-sectional, or case-control designs; non-randomised trials; and secondary research such as systematic reviews or meta-analyses. Records identified in the initial search were screened using EndNote X9.3.3. Study titles and abstracts were independently screened against the inclusion criteria by two reviewers (EM and FT). Records meeting eligibility at the title and abstract stage were advanced to full-text assessment. Discrepancies during screening were resolved through discussion; when consensus was not reached, a third senior reviewer (ED) adjudicated the final decision.

### 2.2. Outcome measures

In this study, the outcomes were categorised into primary and secondary outcomes (Table 1). The primary outcomes consist of sleep quality, sleep efficiency, sleep latency, sleep quantity, theta wave, and delta brain waves. In addition, the secondary outcomes across the included studies were fatigue, anxiety, depression, heart rate, electrodermal response, DVT accuracy, motivation, muscle soreness, inflammatory markers such as CRP, STAI-state and STAI-trait anxiety, HADS anxiety and depression, Chalder Fatigue Scale scores (general, physical, and mental fatigue), salivary chromogranin A, cortisol, perceived stress, dopamine, sleepiness, tenseness, and general mood.

**Table 1.** Summary characteristics of included studies in the review.

Study, year	Country	Study design and duration		Training status	Intervention group	Comparison group	Sleep outcome measure	Primary findings	Secondary findings
**Probiotics**									
Wiącek et al. [18]	Poland	Double-blind RCT, 12 weeks		Dancers	*n* = 5, F, age = 20.00 ± 1.30 y, Lactobacillus helveticus Rosell-52 & Bifidobacterium longum Rosell-17; 3 × 10⁹ CFU/day	*n* = 10, F, age = 20.55 ± 1.04 y, Placebo: Maltodextrin/cornstarch	PSQI: sleep quality (component score 0–3), sleep latency	Sleep quality (PSQI component score, 0 = best to 3 = worst): Trend toward improvement in probiotics group vs. decline in placebo group (PRO: 1.4→1.0; PLA: 0.8→1.2; *p* = 0.0784). Sleep latency: No significant differences (*p* > 0.05).	FAS: Both groups improved over time (PRO: 18.6→12.4; PLA: 15.9→13.1), but there was no significant between-group difference. No significant effect on anxiety or depression was reported.
Adikari et al. [13]	Malaysia	Double-blind RCT, 8 weeks		Football Players	*n* = 10, M, age = 19 ± 0.81 y, Probiotic: Lactobacillus Casei Shirota strain + orange fruit juice (3 × 10¹⁰ CFU/day)	*n* = 9, M, age = 19 ± 0.66 y, placebo: orange fruit juice	EEG (Muse headband): theta and delta wave indices	Week 4: Significant ↑ in theta (relaxation) & delta (attention) brain waves vs. placebo (*p* < 0.05).	Week 8: Significant improvement in reaction time (DVT) vs. placebo (*p* = 0.037). No significant changes in HR, EDR, or DVT accuracy between groups. Suggests possible relaxation & attention benefits with probiotics
Harnett et al., 2020	Australia	Double-blind RCT, 17 weeks		Rugby players	*n* = 9, M, age = 27.0 ± 3.2 y, Ultrabiotic 60™ (60B CFU Lactobacillus, Bifidobacterium, Streptococcus) + Saccharomyces boulardii	*n* = 10, M, age = 26.6 ± 2.9 y, Placebo	Self-report questionnaire (Likert-scale): sleep quality, sleep quantity	Improved sleep quality: Probiotic group showed small–moderate improvement in self-reported sleep quality when muscle soreness decreased and sleep duration & motivation increased.	Correlations: Better sleep quality linked to higher motivation, longer sleep, and lower muscle soreness. Inflammatory markers: Higher CRP = poorer sleep quality; probiotics possibly reduced inflammation’s negative effects on sleep
Sawada et al. [16]	Japan	Double-blind RCT, 12 weeks		University Ekiden runners	*n* = 24, M, age: 19.8 ± 1.4 y, heat-inactivated (non-viable) Lactobacillus gasseri CP2305 preparation, 1 × 10¹⁰ cells/day	*n* = 25, M, age = 20.1 ± 1.1 y, Placebo: isotonic beverage	PSQI (global): sleep quality	Sleep: No significant difference in PSQI global scores (sleep quality) vs. placebo.	Anxiety & Depression: Significant ↓ in STAI-state & STAI-trait anxiety (*p* < 0.05) and HADS anxiety & depression scores (*p* < 0.05) vs. placebo. Fatigue: Significant ↓ in CFS general and physical fatigue scores (*p* < 0.05); trend towards ↓ in mental fatigue scores. Stress Biomarker: Significant ↓ in salivary chromogranin A (stress marker) vs. placebo (*p* < 0.05); no change in cortisol.
**Synbiotics**									
Quero et al. [15]	Spain	Pilot triple-blinded RCT, 4 weeks		Soccer players + sedentary students	*n* = 7 athletes, *n* = 7 sedentary students, M, age = 21.85 ± 1.74 y, Bifidobacterium lactis, Bifidobacterium longum, Lactobacillus rhamnosus + FOS, 1 × 10⁹ CFU/day	*n* = 6 athletes, *n* = 7 sedentary students, M, age = 23.1 ± 3.35 y, Placebo: maltodextrin excipient	Accelerometry (ActiGraph): sleep efficiency, sleep latency; HLPCQ (sleep section): perceived sleep quality	Sleep quality: Significant ↑ in sleep efficiency & latency improvement only in athletes (*p* < 0.05). Perceived stress &	anxiety: Significant ↓ in stress (*p* < 0.01) & anxiety (*p* < 0.05) only in athletes. Depression: Significant ↓ in depression scores in both sedentary & athlete groups (*p* < 0.05). Dopamine: Significant ↑ in dopamine only in athletes (*p* < 0.05), possibly linked to improved mental health
Valle et al. [17]	Brazil	Double-blind RCT, 5 weeks		Military personnel	*n* = 32, M/F, age = 19·69 ± 1.25 y, Lactobacillus acidophilus LA-5: 2.1 × 10⁸ CFU/g; Bifidobacterium animalis BB-12: 2.7 × 10⁹ CFU/g; 2.3 g inulin/60 g serving	*n* = 33, M/F, age = 19.5 ± 1.22 y, Placebo: ice cream	PSQI: sleep quality; questionnaire-based sleepiness	Sleep Quality (PSQI): Both groups improved after 30 d, but only the synbiotic group reached “good sleep quality” (<5 score). Sleepiness ↓ was significantly only in the synbiotic group post-training (*p* < 0.001).	Tenseness: ↓ significantly in the synbiotic group after training (*p* = 0.01) vs. no change in placebo. Mood: General mood scores ↑ post-supplementation then ↓ after training in both groups; no between-group difference except tenseness and sleepiness

Abbreviations: RCT, randomised controlled trial; PSQI, pittsburgh sleep quality index; PRO, probiotic group; PLA, placebo group; CFU, colony-forming units; DVT, digit vigilance test; EDR, electrodermal response; EEG, electroencephalogram; HR, heart rate; FAS, fatigue assessment scale; CFS, chalder fatigue scale; STAI, state–trait anxiety inventory; HADS, hospital anxiety and depression scale; HLPCQ, healthy lifestyle and personal control questionnaire; CRP, C-reactive protein; FOS, fructooligosaccharides; M, male; F, female.

### 2.3. Data extraction

Two separate researchers extracted the data from each selected article (AD and KD). The following information was extracted from each study: first author's name, publication year, country, study design, duration of intervention, subjects' age and sex, training status, type of intervention (probiotics and synbiotics), number of participants, comparison group, and findings of the included studies. In addition, Discrepancies in data extraction were resolved through discussion between the two extractors; if consensus could not be reached, a third senior reviewer (ED) adjudicated.

### 2.4. Risk of bias assessment

Risk of bias in the included studies was independently assessed by two authors (FA and ME) using RoB 2 (the revised Cochrane risk-of-bias tool for randomised trials). RoB 2 evaluates five domains: the randomisation process, deviations from intended interventions, missing outcome data, measurement of the outcome, and selection of the reported result. Judgements within these domains inform an overall rating of “low risk of bias,” “some concerns,” or “high risk of bias” [21]. Additionally, the results of these assessments were visualised using the online robvis tool.

### 2.5. Data analysis

According to the SWiM criteria, the findings of the included articles were narratively synthesised and reported [22]. SWiM was developed to enhance transparency in reporting narrative synthesis, based on systematic reviews that do not include meta-analysis. In this review, meta-analysis was not feasible, even under random-effects assumptions, owing to substantial heterogeneity in supplement interventions, population characteristics, outcome measures, and study designs. Following SWiM guidance, studies were grouped by intervention type (probiotic vs synbiotic) and by sleep outcome domain, including sleep quality, sleep latency, sleep efficiency, sleep quantity, and EEG-derived outcomes. Vote-counting was used based on the direction of effect for each prespecified sleep outcome, using between-group comparisons at the end of the intervention. Outcomes were classified as favouring supplementation if the intervention group improved more than the comparison group, as no effect if there was no between-group difference, and as negative if results favoured control or placebo. Quantitative and qualitative findings were summarised using the direction of effects, the *p*-value for each outcome, and combined *P*-values via Fisher’s method, as implemented in the Corbi packages within R, for each intervention; these results are presented in Table 2. Furthermore, Outcome data were extracted from per-protocol analyses as reported in the included trials. The combined *P*-value was calculated only for studies that reported a significance level. Key study characteristics were qualitatively summarised in Table 1, and quantitative results were tabulated separately. To facilitate transparent presentation of the direction-of-effect synthesis, the results were summarised visually using a harvest-style plot.

**Table 2.** Narrative synthesis for determining the direction of effects, *P*-values of each sleep outcome, and combined *P*-values.

Study, year	Outcome	Positive effect (count)	Negative effect (count)	No effect (count)	*p*-value of each outcome	Combined *p*-value for each intervention type	Combined *p*-value
**Probiotics**							
Wiącek et al. [18],	Sleep latency	0	0	1	p ≥ 0.05	*p* < 0.01	*p* < 0.0001
Wiącek et al. [18],	Sleep quality	1	0	0	p ≥ 0.05		
Adikari et al. [13],	Theta wave	1	0	0	*p* < 0.05		
Adikari et al. [13],	Delta wave	1	0	0	*p* < 0.05		
Harnett et al. 2020	Sleep quality	1	0	0	*p* < 0.05		
Harnett et al. 2020	Sleep quantity	1	0	0	*p* < 0.05		
Sawada et al. [16],	Sleep quality	0	0	1	p ≥ 0.05		
**Synbiotics**							
Quero et al. [15],	Sleep efficiency	1	0	0	*p* < 0.05	*p* < 0.001	
Quero et al. [15],	Sleep latency	1	0	0	*p* < 0.05		
Quero et al. [15],	Sleep quality	0	0	1	NS		
Valle et al. [17],	Sleep quality	1	0	0	*p* < 0.05		
Valle et al. [17],	Sleepiness	1	0	0	*p* < 0.05		

*p-values are presented as significance categories (*p* < 0.05 or *p* ≥ 0.05).

*NS indicates that the p-value was not specified in the source study.

## 3. Results

### 3.1. Study selection and characteristics

**Figure 1.**
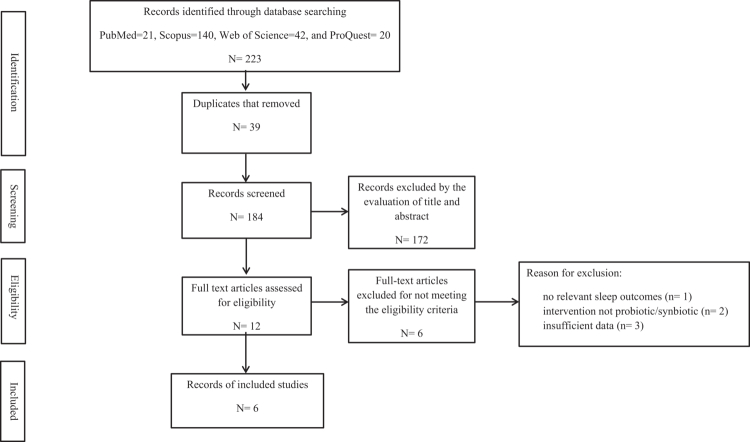
PRISMA 2020 flow diagram of study selection.

Following the removal of duplicates, 184 records were screened, and 172 were excluded based on titles and abstracts. Twelve full-text articles were assessed, of which 6 randomised controlled trials met the eligibility criteria and were included in this review (Figure 1) [13-18]. Additionally, the reasons for excluding the remaining six full-text articles are reported in Figure 1. The included trials comprised four probiotic [13,14,16,18] and two synbiotic [15,17] interventions in exercised populations; intervention duration ranged from 4 to 17 weeks, and publications spanned from 2019 to 2024. All included trials were randomised and blinded. The included articles were five double-blind RCTs [13,14,16-18] and one triple-blinded pilot RCT [15]. Also, trials were conducted in Malaysia [13], Australia [14], Japan [16], Spain [15], Brazil [17], and Poland [18], which they are covering Asia, Europe, South America, and Oceania. In addition, all studies enroled trained populations, including football players [13], rugby players [14], runners [16], female dancers [18], soccer players [15], and military personnel [17]. Four trials were male-only [13-16], one was female-only [18], and one included both sexes [17]. Across studies, sample sizes ranged from *n* = 13 (15) to *n* = 65 [17], with a total of 180 participants across the included RCTs. The characteristics of the included studies are summarised in Table 1.

### 3.2. Primary outcomes

The primary outcomes consist of sleep-related outcomes such as sleep quality (*n* = 5) [14-18], sleep efficiency (*n* = 1)[15], sleep latency (*n* = 2) [15,18], sleep quantity (*n* = 1) [14], theta wave (*n* = 1) [13], and delta brain waves (*n* = 1) [13].

### 3.3. Probiotic supplementation

**Figure 2.**
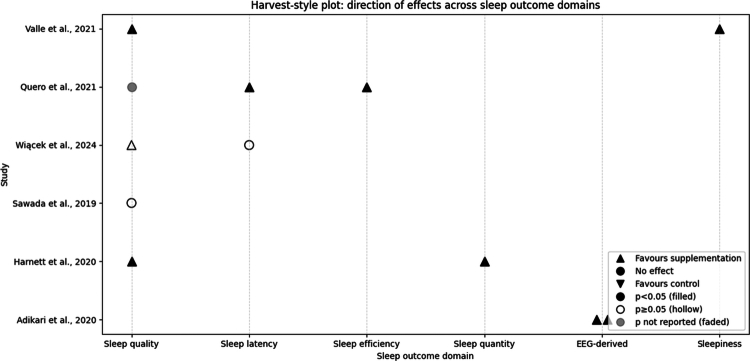
Harvest-style plot summarising the direction of effects across sleep outcome domains in included trials; ▲ indicates effects favouring supplementation, ● no effect, and ▼ effects favouring control, with filled symbols denoting *p* < 0.05, hollow symbols *p* ≥ 0.05, and faded symbols indicating *p*-values not reported.

Significant between-group improvements were observed for theta and delta brain waves at week 4 (*p* < 0.05 for both of them) [13]. Significant improvements were also reported for sleep quality and sleep quantity (both *p* < 0.05) [14]. A positive, non-significant trend was noted for sleep quality (*p* ≥ 0.05) [18], whereas sleep latency (*p* ≥ 0.05) [18] and sleep quality (*p* < 0.05) [16] showed no between-group effect. As mentioned in Table 2, the combined *p*-value for probiotic trials (Fisher’s method) was *p* < 0.01 [13,14,16,18]. Figure 2 presents a harvest-style plot summarising the direction of effects across sleep outcome domains and intervention types.

### 3.4. Synbiotic supplementation

In athletes, sleep efficiency and sleep latency improved significantly (both *p* < 0.05) [15]. In military personnel, sleep quality improved (*p* < 0.05), and sleepiness decreased (*p* < 0.05) [17]. The combined *p*-value for synbiotic trials was *p* < 0.001 [15,17] (Table 2) (Figure 2).

### 3.5. Overall synthesis across sleep outcomes

Pooling all twelve sleep outcomes from six RCTs, the direction of effect favoured supplementation (positive *n* = 9; no-effect *n* = 3; negative *n* = 0). Where exact *p*-values were reported in the source trials, they ranged from 0.009 to 0.418. Fisher’s combined test across all sleep outcomes indicated a significant overall effect, *p* < 0.0001 [13-18] (Table 2).

### 3.6. Secondary outcomes

Regarding secondary outcomes, Wiącek et al. found no significant effects on fatigue, anxiety, or depression [18], while Adikari et al. observed no changes in heart rate, electrodermal response, or DVT accuracy [13]. In one study, better sleep was associated with higher motivation, longer sleep, lower muscle soreness, and reduced inflammation [14]. Also, significant reductions in anxiety, depression, fatigue, and salivary chromogranin A were demonstrated, though cortisol remained unchanged [16]. A decrease in stress, anxiety, and depression, with increased dopamine in athletes, was highlighted [15], and significant improvements in sleepiness and tenseness, alongside transient mood changes, were shown [17].

### 3.7. Quality assessment

**Figure 3.**
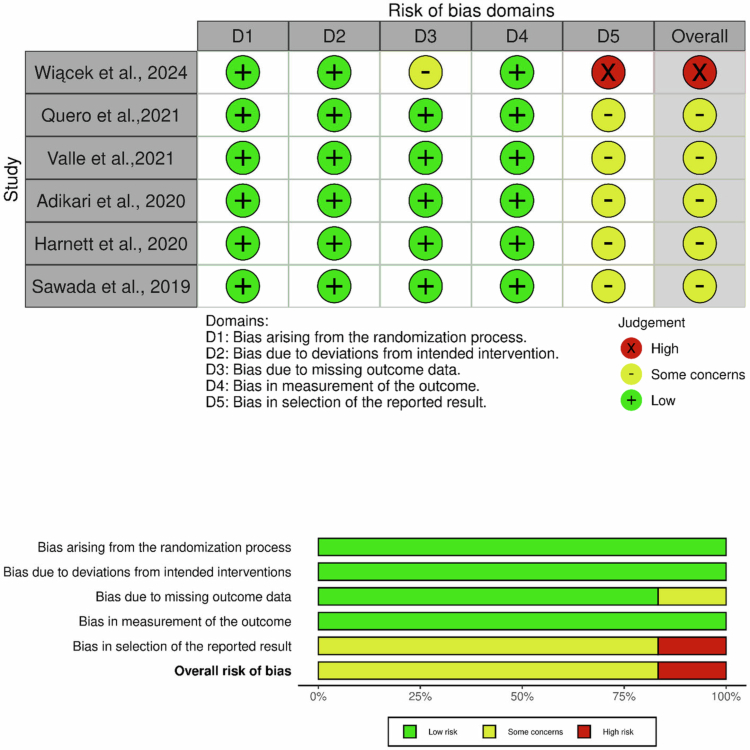
RoB-2 assessments across included RCTs.

Risk of bias was appraised using the RoB 2 tool across five domains (Figure 3). Randomisation and adherence to intended interventions were consistently judged low risk across all trials. Also, Randomisation procedures were not consistently described in the included RCTs. For missing outcome data, judgements were low risk in five trials, with some concerns in one trial [18]. For the measurement of the outcome, all studies were rated low risk. In contrast, selection of the reported result was the principal concern: some concerns were assigned in five trials [13-17], and high risk in one trial (Wiącek et al.) [18]. This was primarily because outcome prespecification was unclear in the five trials rated as ‘some concerns’, while the trial rated as ‘high risk’ showed indications consistent with selective outcome reporting. Overall risk of bias was therefore some concerns in five trials [13-17] and high risk in one trial [18].

## 4. Discussion

This SWiM synthesis indicates that probiotic and synbiotic supplementation tends to improve sleep outcomes in exercised populations, with the weight of evidence favoring supplementation on subjective indices and an absence of adverse signals. Across six randomised, blinded trials, nine of twelve prespecified sleep outcomes moved in a beneficial direction, and Fisher’s combined tests supported an overall effect when narrative aggregation was applied in preference to a conventional meta-analysis. Given the substantial heterogeneity in strains, dosing, duration (4–17 weeks), populations, and sleep endpoints, adopting a SWiM framework was appropriate and increased transparency around grouping rules, standardised summary decisions, and interpretation when quantitative pooling would likely be misleading [22].

Our pattern aligns with broader evidence from non-athletic cohorts showing small but measurable improvements in perceived sleep quality with probiotics. A recent meta-analysis reported reductions in PSQI at both 4–6 and 8–16 weeks, with modest gains in sleep efficiency but inconsistent effects on sleep duration and insomnia severity; importantly, several trials had risk-of-bias or publication-bias concerns that warrant caution when generalising to athletes [12]. Within exercised populations specifically, findings are directionally consistent yet variable in magnitude: in elite rugby union players followed through a competitive season, a multi-species probiotic coincided with favourable changes in self-reported sleep quality alongside reductions in soreness and exploratory links to inflammatory and melatonin dynamics [14]; among professional dancers, trends favoured improved sleep quality but between-group differences did not consistently reach significance, underscoring limited power and population-specific stressors [18]. Synbiotic formulations have shown suggestive benefits in some higher-stress contexts; however, the current evidence is limited, and further trials are needed to confirm this. For example, a triple-blinded pilot in professional soccer suggested improvements in perceived sleep and stress or anxiety, and field training in military personnel showed better sleep quality and reduced sleepiness, although standardised sleep endpoints in such contexts remain sparse [15,17].

Several biological pathways plausibly connect these interventions to sleep. Through the microbiota–gut–brain axis, microbial modulation of tryptophan–serotonin–melatonin flux, short-chain fatty acid signalling, and immune–neuroendocrine tone may influence sleep architecture and circadian regulation [7–9,23,24]. These mechanisms are particularly relevant in sport, where sleep disruption is predictable around intensified training, travel, and competition [5,6]. Exploratory correlations between probiotic use, CRP, and melatonin observed across a season in rugby lend plausibility without establishing causality [14]. In this context, the clustering of benefits on subjective sleep measures is unsurprising: mood, stress, and arousal regulation are proximal to perceived sleep quality, whereas short interventions using wearables or actigraphy may be underpowered to detect small physiological changes, especially amid day-to-day variability typical of training cycles. The general population meta-analysis’ signal at 8–16 weeks further suggests that longer exposures may be necessary to observe more durable or objective changes [12].

### 4.1. Strengths and limitations

Strengths of the evidence include randomised, blinded designs in genuinely trained cohorts spanning multiple sports and stress profiles, which improve ecological validity for performance settings.

Across included trials, the total sample size was small (*n* = 180), limiting precision and generalisability. However, samples are small and predominantly male. Four of the six trials were conducted exclusively in men, with only one female-only trial and one mixed-sex trial; therefore, the generalisability of these findings to female athletes remains uncertain, and interventions vary widely in strain composition and matrix. Interpretation across trials is further limited by differences in product characteristics; notably, one included study used a heat-inactivated non-viable preparation (Sawada et al.), which may not be directly comparable to live probiotic formulations [16]. In addition, intervention durations were relatively short, 4–17 weeks, which may limit the ability to detect durable changes in sleep. Sleep outcomes are heterogeneous, including PSQI subscales, latency/efficiency, spectral EEG features, athlete-specific tools, and device metrics, which constrain comparability and preclude a single pooled effect size. Moreover, outcomes relied largely on subjective sleep measures, while objective sleep assessment, such as actigraphy, was limited, and polysomnography was not used. Risk of bias assessments indicate “some concerns” for selective reporting in several trials and “high risk” in one, and meta-analytic work in the general population highlights potential publication bias [12,14]. These concerns were driven primarily by the RoB 2 domain in the selection of the reported result. In several trials, outcome prespecification was unclear, and one trial was rated high risk due to indications consistent with selective outcome reporting. Together, these issues argue for cautious interpretation of effect size while acknowledging a consistent direction of benefit.

### 4.2. Practical applications

For practice, probiotic or synbiotic supplementation can be considered as an adjunct to established sleep hygiene and scheduling strategies during periods of predictable sleep burden, such as congested fixtures, intensified training blocks, or travel. Expectations should be calibrated: improvements are most likely to manifest in perceived sleep quality and, in some cases, latency, rather than immediate, robust changes in actigraphy-derived duration or efficiency over short cycles. Given strain specificity and the current predominance of multi-species blends, formulations with prior evidence in stress-exposed or athletic contexts are a reasonable starting point, while monitoring both subjective and objective metrics to track response [14,15,17,18]. Also, regarding the limitations of the current evidence base, probiotics and synbiotics should be considered an adjunct rather than a replacement for established sleep strategies.

### 4.3. Future research

Future research in exercised populations should pre-register primary sleep endpoints and adopt a core outcome set that pairs validated questionnaires, such as PSQI or insomnia scales, with objective measures like actigraphy and, where feasible by using polysomnography. To improve consistency across trials, objective outcomes should include actigraphy-derived total sleep time, sleep efficiency, sleep latency, and wake after sleep onset, and assessment windows should be aligned with predictable high-stress periods to reduce noise and enhance sensitivity. Mechanistic markers such as salivary melatonin should be assessed at baseline and at prespecified timepoints during the intervention, with follow-up at approximately four to six weeks and again at the end of the intervention, which is commonly eight to sixteen weeks. Trials should be adequately powered, extend beyond eight to twelve weeks to assess durability, and move toward strain-resolved, dose–response designs, including head-to-head comparisons of probiotic versus synbiotic approaches. Mechanistic integration—microbiome profiling, metabolomics, and endocrine markers such as melatonin and cortisol—will be essential to map causal pathways from microbial modulation to sleep outcomes [7–9,12,14]. Finally, broader sampling across sexes, sport types, and competitive calendars is needed, with reporting that is SWiM-ready (complete statistics, exact *P* values, and transparent outcome hierarchies) to facilitate future synthesis and, ultimately, meta-analysis [21,22].

## 5. Conclusion

In summary, across six randomised trials in exercised populations, probiotic and synbiotic supplementation, limited evidence suggests possible benefits of effect on sleep, most clearly for subjective sleep quality and latency, with fewer and less consistent changes in objective metrics over short durations. Given the small sample sizes, heterogeneity, and risk-of-bias concerns, these findings should be interpreted cautiously. Larger, longer, and strain-resolved trials with standardised sleep outcomes are needed to convert this directional signal into precise, practice-ready guidance [12,14–18,21,22]. Athletes and sports professionals may consider probiotics or synbiotics as an adjunct within a broader sleep-support strategy, with expectations primarily calibrated toward subjective improvements.

## Supplementary Material

Supplementary Material Revised_Supplementary_file_1CLEAN.docx

## Data Availability

The data extracted and analysed during this study are available from the corresponding author upon reasonable request.
