# Supplementary material for: The effect of probiotic and synbiotic supplementation on sleep parameters in exercised population: a systematic review and synthesis without meta-analysis (SWiM) of randomized controlled trials
Source: J Int Soc Sports Nutr. 2026 May 25;23(1):2670564. doi: 10.1080/15502783.2026.2670564 (PMC13202683; doi:10.1080/15502783.2026.2670564)
Supplement: Supplementary Material — Revised_Supplementary_file_1CLEAN.docx [file RSSN_A_2670564_SM1580.docx]

**PICOS, Search Strategy, and Study Selection**

- **PICOS**

| **Search Terms** | **Component** |
| --- | --- |
| Population (P) | “Athletic population” OR “exercised population” |
| Intervention (I) | "Probiotic supplementation” OR “Prebiotic supplementation” OR “Synbiotic supplementation” |
| Comparison (C) | "Standard of care" OR “Control” OR “placebo” |
| Outcome (O) | “Sleep-related parameters” |
| Study Design(S) | “RCT” |

- **This document provides the full search strategy used in this systematic review:**

| athletes | supplementations | sleep | study |
| --- | --- | --- | --- |
| “athlete*” OR “exercise” OR “sport*” OR “trained” OR “training” | “Probiotics” OR “Prebiotics” OR “Lactobacillus” OR “Bifidobacterium” OR “Enterococc” OR “Lactococc” OR “Saccharomyces” OR “Streptococcus salivarius” OR “Streptococcus thermophiles” OR “Streptococcus faecalis” OR “Streptococcus” OR “Synbiotic” OR “Symbiotic” | “sleep” OR “insomnia” OR “circadian rhythm” OR “Polysomnography” OR “PSG” OR “Sleep” OR “Sleep Deprivation” OR “Sleep Quality” OR “Sleep Efficiency” OR “Sleep Stages” OR “Sleep Hygiene” OR “Total sleep time” OR “Sleep Intervention” | "Random Allocation" OR "Single-Blind Method" OR "Double-Blind Method" OR "Cross-Over Studies" OR "Clinical Trials as Topic" OR RCT OR "Intervention Studies" OR intervention OR "controlled trial" OR randomized OR randomised OR random OR randomly OR placebo OR assignment OR trial OR “crossover procedure” OR “equivalence trial” |

| Pubmed | ((("athlete*"[Title/Abstract] OR "exercise"[Title/Abstract] OR "sport*"[Title/Abstract] OR "trained"[Title/Abstract] OR "training"[Title/Abstract]) AND ("Probiotics"[Title/Abstract] OR "Prebiotics"[Title/Abstract] OR "Lactobacillus"[Title/Abstract] OR "Bifidobacterium"[Title/Abstract] OR "Enterococc"[Title/Abstract] OR "Lactococc"[Title/Abstract] OR "Saccharomyces"[Title/Abstract] OR "Streptococcus salivarius"[Title/Abstract] OR "Streptococcus thermophiles"[Title/Abstract] OR "Streptococcus faecalis"[Title/Abstract] OR "Streptococcus"[Title/Abstract] OR "Synbiotic"[Title/Abstract] OR "Symbiotic"[Title/Abstract])) AND ("sleep"[Title/Abstract] OR "insomnia"[Title/Abstract] OR "circadian rhythm"[Title/Abstract] OR "Polysomnography"[Title/Abstract] OR "PSG"[Title/Abstract] OR "Sleep"[Title/Abstract] OR "Sleep Deprivation"[Title/Abstract] OR "Sleep Quality"[Title/Abstract] OR "Sleep Efficiency"[Title/Abstract] OR "Sleep Stages"[Title/Abstract] OR "Sleep Hygiene"[Title/Abstract] OR "Total sleep time"[Title/Abstract] OR "Sleep Intervention"[Title/Abstract])) AND ("Random Allocation"[Title/Abstract] OR "Single-Blind Method"[Title/Abstract] OR "Double-Blind Method"[Title/Abstract] OR "Cross-Over Studies"[Title/Abstract] OR "Clinical Trials as Topic"[Title/Abstract] OR RCT[Title/Abstract] OR "Intervention Studies"[Title/Abstract] OR intervention[Title/Abstract] OR "controlled trial"[Title/Abstract] OR randomized[Title/Abstract] OR randomised[Title/Abstract] OR random[Title/Abstract] OR randomly[Title/Abstract] OR placebo[Title/Abstract] OR assignment[Title/Abstract] OR trial[Title/Abstract] OR "crossover procedure"[Title/Abstract] OR "equivalence trial"[Title/Abstract]) | 21 |
| --- | --- | --- |
| Web of Science | “athlete*” OR “exercise” OR “sport*” OR “trained” OR “training” (Topic) and “Probiotics” OR “Prebiotics” OR “Lactobacillus” OR “Bifidobacterium” OR “enterococci” OR “lactococci” OR “Saccharomyces” OR “Streptococcus salivarius” OR “Streptococcus thermophiles” OR “Streptococcus faecalis” OR “Streptococcus” OR “Synbiotic” OR “Symbiotic” (Topic) and “sleep” OR “insomnia” OR “circadian rhythm” OR “Polysomnography” OR “PSG” OR “Sleep” OR “Sleep Deprivation” OR “Sleep Quality” OR “Sleep Efficiency” OR “Sleep Stages” OR “Sleep Hygiene” OR “Total sleep time” OR “Sleep Intervention” (Topic) and "Random Allocation" OR "Single-Blind Method" OR "Double-Blind Method" OR "Cross-Over Studies" OR "Clinical Trials as Topic" OR RCT OR "Intervention Studies" OR intervention OR "controlled trial" OR randomized OR randomised OR random OR randomly OR placebo OR assignment OR trial OR “crossover procedure” OR “equivalence trial” (Topic) \| 42 results | 42 |
| Scopus | ( TITLE-ABS-KEY ( "athlete*" OR "exercise" OR "sport*" OR "trained" OR "training" ) AND TITLE-ABS-KEY ( "sleep" OR "insomnia" OR "circadian rhythm" OR "Polysomnography" OR "PSG" OR "Sleep" OR "Sleep Deprivation" OR "Sleep Quality" OR "Sleep Efficiency" OR "Sleep Stages" OR "Sleep Hygiene" OR "Total sleep time" OR "Sleep Intervention" ) AND TITLE-ABS-KEY ( "Probiotics" OR "Prebiotics" OR "Lactobacillus" OR "Bifidobacterium" OR "Enterococc" OR "Lactococc" OR "Saccharomyces" OR "Streptococcus salivarius" OR "Streptococcus thermophiles" OR "Streptococcus faecalis" OR "Streptococcus" OR "Synbiotic" OR "Symbiotic" ) AND TITLE-ABS-KEY ( "Random Allocation" OR "Single-Blind Method" OR "Double-Blind Method" OR "Cross-Over Studies" OR "Clinical Trials as Topic" OR RCT OR "Intervention Studies" OR intervention OR "controlled trial" OR randomized OR randomised OR random OR randomly OR placebo OR assignment OR trial OR "crossover procedure" OR "equivalence trial" ) ) | 140 |
| ProQuest | noft(“athlete*” OR “exercise” OR “sport*” OR “trained” OR “training”) AND noft(“Probiotics” OR “Prebiotics” OR “Lactobacillus” OR “Bifidobacterium” OR “Enterococc” OR “Lactococc” OR “Saccharomyces” OR “Streptococcus salivarius” OR “Streptococcus thermophiles” OR “Streptococcus faecalis” OR “Streptococcus” OR “Synbiotic” OR “Symbiotic”) AND noft(“sleep” OR “insomnia” OR “circadian rhythm” OR “Polysomnography” OR “PSG” OR “Sleep” OR “Sleep Deprivation” OR “Sleep Quality” OR “Sleep Efficiency” OR “Sleep Stages” OR “Sleep Hygiene” OR “Total sleep time” OR “Sleep Intervention”) AND noft("Random Allocation" OR "Single-Blind Method" OR "Double-Blind Method" OR "Cross-Over Studies" OR "Clinical Trials as Topic" OR RCT OR "Intervention Studies" OR intervention OR "controlled trial" OR randomized OR randomised OR random OR randomly OR placebo OR assignment OR trial OR “crossover procedure” OR “equivalence trial”) | 20 |
| ALL |  | 223 |
| Duplicates |  | 39 |
| reminds |  | 184 |

*The literature search was conducted on **16 September 2025** across all selected databases.

- **Inclusion and Exclusion Criteria**

**Inclusion Criteria:**

(a) to be an RCT

(b) to investigate the athletic population or the exercised population

(c) to be an original study looking for any short- or long-term effects imposed by any type of probiotic, prebiotic, or synbiotic supplements

(d) to report sufficient data about parameters related to sleep as a primary or secondary outcome

**Exclusion Criteria:**

(a) in vitro, in silico, or in vivo animal studies

(b) ecological/ cross-sectional/ case-control studies, non-randomized trials, systematic reviews, and meta-analyses
